# Foetal defence against cancer: a hypothesis

**DOI:** 10.1111/jcmm.12095

**Published:** 2013-07-01

**Authors:** Hui-Tai Yang, Kuo-Ching Chao

**Affiliations:** a Department of Internal Medicine, Taipei City HospitalTaipei, Taiwan; b Department of Internal Medicine School of Medicine College of Medicine, Taipei Medical UniversityTaipei, Taiwan; c Department of Internal Medicine, Taipei Medical University HospitalTaipei, Taiwan

It was once believed that the placenta blocks direct cell transfer between the mother and the foetus, that is, until the discovery of the maternal and foetal microchimerism which proved the existence of cell trafficking during pregnancy [1, 2]. It has been reported that 100% of the pregnant women at 36th week carry foetal cells in their circulation, the prevalence of which decreases, by 22–75%, after child delivery. The foetal cells found in maternal tissues include cells of mesenchymal and hematopoietic origins, T cell, B-cells and NK-cells, *etc*. Similarly, some maternal cells, such as the lymphoid and myeloid cells, T cells, B-cells, monocyte/macrophages and NK-cells, have been detected in some umbilical cord blood and in a number of young adults.

As cell transfer is possible between mother and foetus, it is highly conceivable that the mother's cancer cells could pass through the placenta to reach the foetus as well. Interestingly enough, statistical data [3–6] showed that in 98 cases of pregnant women with cancer, placental metastasis were noted in 90 cases (91.84%), but foetal metastasis only in 17 cases (17.35%). Among the 90 cases, in those diagnosed with breast cancer (14 cases), ovarian cancer (two cases), and malignant sarcoma (eight cases), although metastatic spread to the placenta was confirmed, no metastasis in the foetuses was found. In addition, in the cases of malignant melanoma, lung cancer, leukaemia and lymphoma, the percentages of placental metastasis were high, but the percentages were relatively low for foetal metastasis. Therefore, based on the above findings, it has been concluded that during pregnancy there must be a defence mechanism blocking the metastasis of these harmful cancer cells to the foetuses. The question is, which cell, or cells, plays this role?

Histologically, maternal and foetal circulations are separated by three components: the trophoblast, the villous connective tissue and the capillary wall. Some reports indicated that probably the trophoblast plays the role of a physical barrier in recognizing and rejecting foreign maternal antigens. Phagocytosis and destruction of tumour cells by the villous syncytiotrophoblast and the villous trophoblast have also been reported [7, 8]. Moreover, it was observed that once the invasion of cancer cells into the chorionic villous takes place, there is almost no avoidance of the foetal metastasis [3, 9]. Hence, the question worth digging into: What happens when the cancer is of the trophoblast origin?

Gestational choriocarcinoma is a highly malignant trophoblastic neoplasm developed during pregnancy, and intraplacental choriocarcinoma is a such type of gestational cancer grown in the placenta that is usually not identified until maternal metastasis has taken place. Review of the literature [10] showed that of 11 cases of intraplacental choriocarcinoma with maternal metastasis, on top of two that were lost to stillbirth, only two were noted to have foetal metastasis. This meant that seven of the 11(63.64%) foetuses were spared of the metastasis of the disease, which also could mean, in cancer of the trophoblast origin, metastasis to the immunogically naïve foetus is still a rarely occurrence despite the maternal metastasis. Therefore, this could suggest that, in addition to the trophoblast, there should be another defence mechanism in the area of placenta or umbilical cord that blocks the trafficking of the cancer cells from the placenta to the foetus.

Wharton's Jelly is the primitive connective tissue of the umbilical cord lying between the amniotic epithelium and the umbilical vessels. The main role of the Wharton's Jelly is to protect the umbilical vessels from compression, torsion and bending. Wharton's Jelly cells (WJCs), also known as the human umbilical cord mesenchymal stem cells (HUMSCs), are cells isolated from the Wharton's Jelly. Wharton's Jelly cells are characterized by their self-renewal and multipotency [11–13]. They showed a great *in vitro* and *in vivo* plasticity, towards lineages such as the hepatocytes [14], pancreatic beta cells [13, 15] and cardiomyocytes [16]. They are also able to support the stem cell niche [17] and synthesize various cytokines [17], and they possess the properties of immunomodulation [18] and homing [19]. Researchers postulated that these mesenchymal stromal cells are likely the cells trapped in the connective tissue matrix during their migration to and from the placenta through the developing umbilical cord during early embryogenesis and remain there for the duration of gestation [20]. It was noted that WJCs not only possess MSC properties but they exhibit properties similar to those attributed to embryonic stem cell (ESC) as well [21]. However, it is still less obvious whether WJC plays a role during embryonic and foetal development.

In the literature, MSCs can either suppress or promote tumours. Recently, it was found that culturing human bone marrow mesenchymal stem cells (HBMSCs) with tumour necrosis factor-α (TNF-α) enhanced their tumour-suppressive properties through the upregulation of multiple genes with cancer apoptotic activity. The HBMSCs preactivated with TNF-α induced apoptosis in MDA-MB231 breast cancer cells, suppressed MDA-MB231 cell cycling, and inhibited the progression of tumours formed from MDA-MB231 [22]. As for WJCs, they are described as potent immunomodulatory cells and new molecules are discovered *in vitro* almost weekly. One of the more promising molecules is represented by B7-H3, a member of the B7 co-stimulator family. This molecule has been linked to both pro-tumorigenic and antitumor activities [23]. Recent data showed that this molecule, which is not expressed in BM-MSCs, is expressed in WJCs both when kept undifferentiated and in their differentiated progeny [16, 24]. Another set of molecules with importance in the immunomodulatory function of WJC are non-classical HLAs (*e.g*. HLA-E and HLA-G). Both were related to cancer progression or immune evasion by a number of studies [25], and their expression was demonstrated in WJCs by different groups [26, 27].

**Fig. 1:**
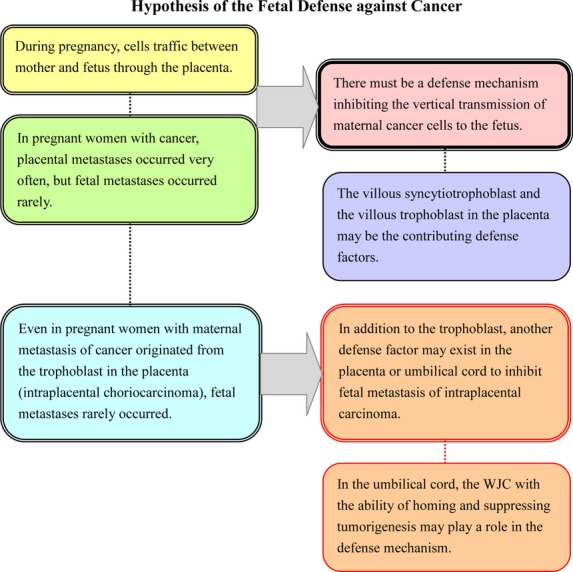
Hypothesis of the fetal defense against cancer.

In a previous study [28], we reported the interactions between selected WJC (HUMSC) and MDA-MB231 which caused MDA-MB231 breast cancer cell death, include (1) binding mechanism: breast cancer cell apoptosis from direct cell-cell contact with WJC and infusion of some substance into cancer cell by WJC; (2) cell-in-cell mechanism (a novel phenomenon we named ‘cic-apoptosis’): breast cancer cell apoptosis following forming of a cell-in-cell structure of WJC internalized within cancer cell; (3) indirect (cytokine) mechanism: attenuation of breast cancer cell growth from one or more cytokines secreted, predominantly, by co-cultured WJC and MDA-MB231 or by WJC alone, without direct contact with cancer cells. The WJC was proved to have the ability of homing and suppressing tumorigenesis [28–30] both *in vitro* and *in vivo*. Therefore, we can make a bold assumption that, in addition to the trophoblast in the placenta, WJC in the umbilical cord also plays a role in the foetal defence against the invasion of maternal or placental cancer cells. In the event of cancer cells occurrence in the placenta, WJCs may home to the site and induce apoptosis of the cancer cells. We invite further investigations that are much needed to help substantiate our hypothesis which states that WJCs may not just be the cells accidentally embedded in the Wharton's Jelly during embryogenesis but are cells purposely placed there as an essential guard in the umbilical cord during foetal development. Moreover, the WJC induced apoptosis of cancer cells, different from cell necrosis, does not cause severe inflammation, and that may shed light on cell therapy for cancer in the future.
